# Antibiofilm and Antivirulence Properties of Indoles Against *Serratia marcescens*

**DOI:** 10.3389/fmicb.2020.584812

**Published:** 2020-10-30

**Authors:** Sivasamy Sethupathy, Ezhaveni Sathiyamoorthi, Yong-Guy Kim, Jin-Hyung Lee, Jintae Lee

**Affiliations:** School of Chemical Engineering, Yeungnam University, Gyeongsan, South Korea

**Keywords:** biofilm, indoles, motility, protease, prodigiosin, quorum sensing, *S. marcescens*

## Abstract

Indole and its derivatives have been shown to interfere with the quorum sensing (QS) systems of a wide range of bacterial pathogens. While indole has been previously shown to inhibit QS in *Serratia marcescens*, the effects of various indole derivatives on QS, biofilm formation, and virulence of *S. marcescens* remain unexplored. Hence, in the present study, we investigated the effects of 51 indole derivatives on *S. marcescens* biofilm formation, QS, and virulence factor production. The results obtained revealed that several indole derivatives (3-indoleacetonitrile, 5-fluoroindole, 6-fluoroindole, 7-fluoroindole, 7-methylindole, 7-nitroindole, 5-iodoindole, 5-fluoro-2-methylindole, 2-methylindole-3-carboxaldehyde, and 5-methylindole) dose-dependently interfered with quorum sensing (QS) and suppressed prodigiosin production, biofilm formation, swimming motility, and swarming motility. Further assays showed 6-fluoroindole and 7-methylindole suppressed fimbria-mediated yeast agglutination, extracellular polymeric substance production, and secretions of virulence factors (e.g., proteases and lipases). QS assays on *Chromobacterium violaceum* CV026 confirmed that indole derivatives interfered with QS. The current results demonstrate the antibiofilm and antivirulence properties of indole derivatives and their potentials in applications targeting *S. marcescens* virulence.

## Introduction

*Serratia marcescens* is a Gram-negative, rod-shaped bacterium that belongs to the *Enterobacteriaceae* family and well-known for its ability to produce prodigiosin a red pigment (Williamson et al., 2006). The bacterium is often isolated from clinical samples and is ubiquitous in nature. During the past four decades, *S. marcescens* has been increasingly recognized as an important opportunistic pathogen that has developed resistance to many antibiotics and is frequently associated with nosocomial infections in pediatric, adult, aged, and immunocompromised patients. *S. marcescens* causes surgical site, eye, respiratory tract, bloodstream, urinary tract, urinary catheter-associated, and gastrointestinal tract infections and endocarditis, meningitis, and other diseases (Cristina et al., 2019). It has been estimated that 6.5% of Gram-negative bacterial pathogenic infections are caused by *Serratia* spp. in the United States and Europe (Sader et al., 2014). *Serratia* spp. is also known to be the 7th and 10th most common cause of pneumonia and bloodstream infections, respectively, in United States and Europe (Acar and Goldstein, 1997; Jones, 2010). *S. marcescens* isolates of clinical origin have been shown to produce extended-spectrum-β-lactamase (Yang et al., 2012), and to acquire multiple drug resistance via horizontal gene transfer from other members of the *Enterobacteriaceae* family (Ivanova et al., 2008). The bacterium is known to utilize a broad array of nutrients and to grow in the presence of antiseptics, detergents, and disinfectants (Cooney et al., 2014).

Quorum sensing (QS) in *S. marcescens* plays important roles in antibiotic resistance, biofilm formation, synchronizing the productions of proteases, lipases, prodigiosin, and butanediol, and swimming and swarming motilities (Van Houdt et al., 2007). *S. marcescens* strains produce a wide range of *N*-acylhomoserine lactones (AHLs) (e.g., C4-AHL, C6-AHL, 3-oxo-C6-AHL, C7-AHL, and C8-AHL), which it uses as QS signal molecules (Eberl et al., 1996; Horng et al., 2002; Coulthurst et al., 2006). Furthermore, biofilms formed by *S. marcescens* clinical isolates are resistant to commonly used antibiotics (Ray et al., 2017), and biofilm formation by clinically important bacterial pathogens is primarily responsible for device-associated chronic infections. QS defective mutants have been reported to be less virulent and incapable of forming robust biofilms, and thus, QS inhibition is a strategy used to control the virulence of *S. marcescens* (LaSarre and Federle, 2013). Several bioactive compounds, including alpha-bisabolol and vanillic acid, have been successfully used to inhibit virulence factor production and biofilm formation by *S. marcescens* (Sethupathy et al., 2016b, 2017).

In biofilms, bacterial cells are surrounded by a self-secreted polymeric matrix comprised of macromolecules such as proteins, lipids, carbohydrates, and extracellular DNA, which protect the bacterium from environmental stress factors, disinfectants, host immune system, and antibiotics (Stewart and Costerton, 2001; Høiby et al., 2010). Bacterial cells in biofilms are metabolically less active and grow more slowly and this characteristic facilitates their acquisition of antibiotic resistance (Stewart, 2002). Hence, it appears suitable combinations of biofilm/QS inhibitors and conventional antibiotics might usefully enhance the antibiotic susceptibilities of bacterial cells in biofilms (Brackman et al., 2011).

It has been reported that more than 85 species of Gram-positive and Gram-negative bacteria can synthesize indole (Lee and Lee, 2010). Indole is an important bacterial signal molecule that regulates several important biological processes such as genetic stability, metabolism, biofilm formation, pathogenesis, antibiotic resistance, and oxidative stress responses and also acts as an interspecies and interkingdom signal to regulate diverse functions (Lee et al., 2015b). Several studies have described the antibiofilm and antivirulence activities of indole derivatives (e.g., 7-hydroxyindole, 3-indoleacetonitrile, 7-fluoroindole, 7-benzyloxyindole, and methylindoles) against clinically important pathogens, such as enterohemorrhagic *Escherichia coli* (Lee et al., 2007), *Pseudomonas aeruginosa* (Lee et al., 2009, 2011, 2012), *Staphylococcus aureus* (Lee et al., 2013), and *Candida albicans* (Lee et al., 2018; Manoharan et al., 2018). In addition, indole and 3-indolylacetonitrile have been shown to inhibit the maturation of *Paenibacillus alvei* endospores (Kim Y.-G. et al., 2011), and halogenated indoles have been reported to have nematicidal and insecticidal potentials (Rajasekharan et al., 2019, 2020). Although the QS inhibitory activity of indole in *S. marcescens* has been clearly reported in previous work (Hidalgo-Romano et al., 2014), the effects of indole derivatives on the biofilm and virulence of indole-negative *S. marcescens* have yet to be evaluated, and thus in the present study, we investigated the antibiofilm and antivirulence potentials of several indole derivatives against *S. marcescens*.

## Materials and Methods

### Indole Compounds

Indole and 50 indole derivatives (Supplementary Figure 1) were purchased from Sigma-Aldrich (St. Louis, MO, United States) and Combi-Blocks, Inc. (San Diego, CA, United States), dissolved in dimethyl sulfoxide (DMSO) to produce 1 M stock solutions, and stored at −20°C. DMSO (0.1% v/v) was used as the negative control and at the concentrations present (<0.1%) did not affect bacterial growth or biofilm formation.

### Bacterial Culture Conditions

*S. marcescens* ATCC 14756 was streaked on Luria-Bertani (LB) agar and incubated at 30°C for 24 h. Plates were stored at 4°C until required. For biofilm and other assays, a single colony of *S. marcescens* was inoculated in LB broth and cultured at 30°C for 12 h at 160 rpm. Two percentage of the overnight culture (adjusted to 0.5 McFarland containing ∼1 × 10^8^ CFU mL^–1^) was used as an inoculum for biofilm and other virulence assays.

### Prodigiosin Assay

*S. marcescens* was grown in the absence or presence of indole or indole derivatives at 30°C for 20 h and centrifuged at 12,000 rpm for 10 min. Acidified ethanol (1 mL of 4% 1 M HCl in ethanol) was added to the cell pellets obtained and vortexed vigorously to extract prodigiosin. After centrifugation, supernatant absorbances were measured at 534 nm as previously reported (Slater et al., 2003).

### Biofilm Inhibition Assay

*S. marcescens* cells were inoculated in 2 mL LB and incubated at 30°C for 20 h. To assess biofilm inhibitory activity, cells were re-inoculated into LB (dilution ratio 1:50) and cultured overnight in 96-well plates in the absence or presence of indole or indole derivatives under static conditions at 30°C for 20 h. After incubation, planktonic cell densities were measured at 620 nm and culture supernatant were discarded. Residual planktonic cells were removed by washing plates three times with water. Biofilms that formed on the plates were stained with crystal violet (0.1%) for 20 min, excess dye was removed by washing three times, and bound crystal violet was solubilized in 95% ethanol. Absorbances were measured at 570 nm using a Multiskan EX microplate photometer (Thermo Fisher Scientific, Waltham, MA, United States) (Lee et al., 2011).

### Growth Curve Analysis and Determination of Minimum Inhibitory Concentrations (MICs)

*S. marcescens* was grown in the absence or presence of indole or selected indole derivatives, as described above, and planktonic cell growth was monitored periodically at 600 nm for 24 h using an Optizen 2120UV spectrophotometer (Mecasys Co., Ltd., Daejeon, Korea). MIC was defined as the lowest concentration that inhibited planktonic cell growth by 80% and also confirmed by colony counting. MICs were determined for 3-indoleacetonitrile, 5-fluoro-2-methylindole, 5-fluoroindole, 6-fluoroindole, 5-methylindole, 7-methylindole, and indole as previously described (Lee et al., 2013).

### Microscopic Observation of Biofilms

*S. marcescens* cells were inoculated in 2 mL of LB and incubated at 30°C for 20 h. Cells were then re-inoculated in LB at a dilution ratio of 1:50, cultured overnight in 96-well plates with indole, 6-fluoroindole, or 7-methylindole at 1 mM, and then incubated at 30°C for 24 h without shaking. For confocal laser scanning microscope (CLSM) analysis, cells were stained with 100 μL of pre-warmed PBS containing carboxyfluorescein diacetate succinimidyl ester for 20 min at 30°C (final concentration, 5 μM) and then washed with PBS (Lee et al., 2011). Cells were visualized by a CLSM (Nikon Eclipse Ti, Tokyo, Japan) using a 20× objective and an Ar laser (excitation wavelength 488 nm, emission wavelength range 500–550 nm). In each experiment, at least 10 random positions in three independent cultures were chosen for microscopic analysis.

### Swarming and Swimming Assay

Swimming agar (1% peptone, 0.5% NaCl, and 0.3% agar) and swarming agar plates (1% peptone, 0.5% NaCl, and 0.6% agar) were prepared with or without indole (0.5 or 1 mM) or selected indole derivatives. Plates were spotted with 5 μL overnight culture of *S. marcescens*, incubated in an upright position at 30°C for 16 h, and then swimming and swarming motility inhibitions were assessed as previously described (Pearson, 2019).

### Protease Assay

Extracellular casein degrading protease activities of supernatants from *S. marcescens* grown in the absence or presence of indole or indole derivatives were measured using 2% w/v of azocasein. Briefly, equal volumes of azocasein and culture supernatants were reacted at 37°C for 30 min and then 600 μL of 10% trichloroacetic acid was added to stop the proteolysis. Reaction tubes were kept for 30 min at −20°C to precipitate unreacted azocasein and centrifuged. An equal volume of 1 M NaOH was added to supernatants (700 μL), and absorbances were read at 440 nm as previously described (Coulthurst et al., 2006).

### Lipase Assay

The effects of indole and indole derivatives on extracellular lipase production were evaluated by incubating 1 volume of supernatant from a *S. marcescens* control with 9 volumes of substrate buffer [1 volume of buffer A containing 3 mg/mL of *p*-nitrophenyl palmitate in isopropyl alcohol, 9 volumes of buffer B containing 1 mg/mL of gummi arabicum and 2 mg/mL sodium deoxycholate in 50 mM Na_2_PO_4_ buffer (pH-8.0)] for 30 min in the dark at room temperature. After incubation, reaction tubes were centrifuged at 12,000 rpm for 10 min and lipase activity was terminated by adding 1 volume of 1 M Na_2_CO_3_. Absorbances was measured at 405 nm as previously described (Patel et al., 2018).

### Fimbria Activity Assay

Effects of indole and indole derivatives on *S. marcescens* fimbria activity were accessed using *Saccharomyces cerevisiae* (Sigma, product no. YSC2), as previously described (Shanks et al., 2007). Yeast agglutination was measured spectrophotometrically by adding 1.5 mL of PBS containing 0.5 mL of *S. cerevisiae* (2% w/v in PBS) and 0.4 mL of *S. marcescens* cells in PBS (OD_600_, 0.5). To achieve a uniform mixture of *Saccharomyces cerevisiae* and *Serratia marcescens* cell suspension, the reaction tubes were gently vortexed for 5 s and the initial OD_600_ was measured. After 10 min of incubation at room temperature, 100 μL of upper phase was transferred to a 96 well plate and OD_600_ was measured. During 10 min incubation, fimbria present on the *S. marcescens* cell binds with *S. cerevisiae* and agglutinates *S. cerevisiae*. Formation of agglutination indicates the presence of fimbria on *S. marcescens* cells and a decrease in the fimbria results in the reduction of aggregate formation. Presence of visible aggregates of agglutinated cells affected the OD_600_ measurement and hence vigorous vortexing for 30 s was done to disturb the agglutinated cells before the reading of the second OD_600_ values. Percentage agglutination was calculated using 100 × (1-OD_600_ before vortexing/OD_600_ after vortexing).

### H_2_O_2_ Sensitivity Assay

Disk diffusion assays were performed by spreading control and treated cells on LB agar plates, placing 6-mm sterile paper disks on the agar, loading disks with 10 μL of 30% H_2_O_2_, and then incubating plates for 24 h at 30°C. Zones of inhibition were measured as previously described (Shanks et al., 2007).

### Extracellular Polymeric Substance (EPS) Extraction and FTIR

Extracellular polymeric substance (EPS) extraction was carried as previously described (Badireddy et al., 2008). Briefly, 100 mL of *S. marcescens* control and 6-fluoroindole or 7-methylindole (1 mM) treated cultures for 24 h were centrifuged at 7,000 rpm for 15 min at 4°C to collect cells. Cell-free culture supernatants were stored at −20°C for cell-free EPS extraction. Collected cell pellets were washed with wash buffer (10 mM Tris/HCl pH 8.0, 10 mM EDTA), resuspended in 100 mL of isotonic extraction buffer (10 mM Tris/HCl pH 8.0, 10 mM EDTA, 2.5 mM NaCl), incubated at 4°C for 12 h, vortexed for 5 min, and centrifuged at 5,000 rpm for 15 min to collect supernatants containing cell bound EPS. Cell-free culture supernatants containing cell free EPS and isotonic buffer containing cell bound EPS were pooled, mixed with 3 volumes of ice-cold ethanol, and kept at −20°C for 18 h to precipitate EPS. Precipitated EPS was collected by centrifugation at 10,000 rpm for 10 min at 4°C, vacuum dried, and analyzed by FTIR spectrometry (Spectrum Two^TM^ FTIR, PerkinElmer, Massachusetts, United States) at 400–4,000 cm^–1^.

### RNA Isolation and Transcriptomic Studies

For transcriptomic analyses, 25 mL of *S. marcescens* at an initial turbidity of 0.05 at OD_600_ was inoculated into LB broth in 250 mL Erlenmeyer flasks and incubated for 6 h at 30°C with agitation at 250 rpm in the presence or absence of 6-fluoroindole (0.5 mM). To prevent RNA degradation, RNase inhibitor (RNAlater, Ambion, TX, United States) was added to cells immediately after incubation. Total RNA was isolated using a hot acidic phenol method (Amin-ul Mannan et al., 2009), and RNA was purified using a Qiagen RNeasy mini Kit (Valencia, CA, United States).

Quantitative reverse transcriptase PCR (qRT-PCR) was used to determine the expressions of 10 QS-related genes list all *bmsA* (biofilm), *carA* (prodigiosin production), *fimA* (type 1 fimbriae), *flhD* (motility), *luxS* (quorum sensing), *pigA* (prodigiosin production), *pigC* (prodigiosin production), *Sma*I*/R* (LuxIR-type quorum sensing system), and *rpoS* (motility and biofilm). The specific primers and housekeeping gene (*16S rRNA*) used for qRT-PCR are listed in Supplementary Table 1. The expression of *16S rRNA* was not affected by 6-fluoroindole. The qRT-PCR method used was as described by Kim Y.-G.et al. (2016), and was performed using SYBR Green master mix (Applied Biosystems, Foster City, United States) and an ABI StepOne Real-Time PCR System (Applied Biosystems). At least two independent cultures with four repetitions were used.

### Quorum Sensing Inhibition Assay

Quorum sensing (QS) inhibition was assayed as previously described (Kim Y.-G. et al., 2015). *Chromobacterium violaceum* CV026 is deficient in QS signal production, and thus, cannot produce the purple pigment violacein. However, pigment production can be restored by the exogenous addition of AHLs [*N*-butanoyl-L-homoserine lactone (BHL) or *N*-hexanoyl homoserine lactone (HHL) at 500 μM]. An overnight culture of CV026 was diluted with fresh LB broth (1:20), aliquoted (300 μL) into 96-well polystyrene microtiter plate, and treated with indoles. Mixtures were incubated at 30°C for 2 days.

### Statistical Analysis

Most assays were conducted with two independent cultures with six repetitions while motility, EPS quantification, and qRT-PCR assays were performed with two independent cultures with four repetitions. Results are expressed as means ± standard deviations. The student’s *t*-test was used to determine the significances of intergroup differences, and statistical significance was accepted for *P* < 0.05.

## Results

### Effects of Indole Derivatives on Prodigiosin Production, Biofilm Formation, and Swarming and Swimming Motilities

**FIGURE 1:**
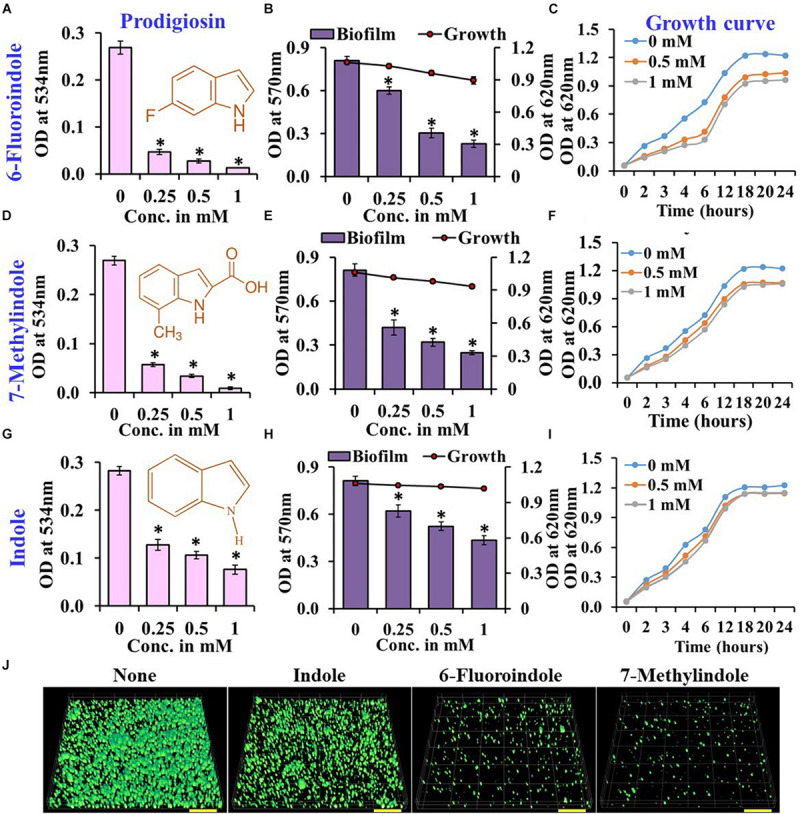
Effects of indole, 6-fluoroindole, or 7-methylindole on prodigiosin production **(A,D,G)**, 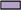 biofilm formation and 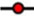 planktonic cell growth **(B,E,H)**, and the 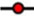 planktonic cell growth **(C,F,I)** of *S. marcescens*. Error bars represent standard deviations. CLSM images of *S. marcescens* biofilms formed in the presence or absence of 1 mM indole, 6-fluoroindole, and 7-methylindole **(J)**. Scale bars represent 100 μM. Error bars and asterisks (*) represent standard deviations and statistically significant differences (*p* < 0.05), respectively, vs. non-treated controls.

The quorum sensing inhibitory activities of the 51 indole derivatives were assessed by measuring their abilities to inhibit prodigiosin production by *S. marcescens*. Among the indole derivatives tested, 2-oxindole, 5-fluoroxindole, 3-indoleacetonitrile, 5-fluoroindole, 6-fluoroindole, 7-fluoroindole, 5-fluoroindole-2,3-dione, 7-methylindole, 7-nitroindole, 7-azaindole, 5-iodoindole, 5-fluoro-2-methylindole, 5-chloro-2-methylindole, 5-indoindolin-2-one, indole-3-acetamide, and 5-methylindole at 1 mM reduced prodigiosin production by 50–80% (Supplementary Figure 1). Additional assays with 3-indoleacetonotrile, 5-fluoro-2-methylindole, 5-fluoroindole, 6-fluoroindole, 5-methylindole, and 7-methylindoles demonstrated concentration-dependent reductions in prodigiosin production (Figures 1A,D,G and Supplementary Figure 2). These inhibitions suggested the above mentioned indole derivatives interfere with the QS system in *S. marcescens.*

In our previous studies, we demonstrated the antibiofilm activities of indole derivatives, such as 7-hydroxyindole (Lee et al., 2009), 3-indoleacetonitrile, indole-3-carboxyaldehyde (Lee et al., 2011), 7-fluoroindole (Lee et al., 2012), 5-iodoindole (Lee et al., 2016), and methylindoles (Lee et al., 2018). In the current study, indole derivatives such as 5-fluoroxindole, 3-indoleacetonitrile, 5-fluoroindole, 6-fluoroindole, 7-methylindole, 7-nitroindole, 5-chloro-2-methylindole, 7-methylindole-3-carboxaldehyde, 5-methylindole, indole-3-acetamide, indole-3-propionic acid, 4-benzyloxyindole and 5-benzyloxyindole inhibited biofilm formation by *S. marcescens* by 40–75% (Figures 1B,E,H and Supplementary Figure 3). Particularly, 3-indoleacetonitrile, 5-fluoro-2-methylindole, 5-fluoroindole, 6-fluoroindole, 5-methylindole, 7-methylindole, 5-iodoindole, and indole dose-dependently reduced both biofilm formation and prodigiosin production (Figure 1 and Supplementary Figures 1–4). CLSM analysis confirmed the biofilm inhibitory activities of these two indole derivatives and indole as evidenced by obvious reductions in surface coverage and biomass in biofilms (Figure 1J).

Growth curve analysis was used to evaluate the effects of these six derivatives and indole on the growth of *S. marcescens*. The obtained results revealed 6-fluoroindole and 7-methylindole had slight bacteriostatic activity but not in the presence of indole up to 1 mM (Figures 1C,F,I). Growth curve analysis results for *S. marcescens* grown in the presence or absence of 3-indoleacetonitrile, 5-fluoro-2-methylindole, 5-fluoroindole, or 5-methylindole are presented in Supplementary Figure 5. We also found the MICs of 6-fluoroindole (Figure 3A), 7-methylindole (Figure 3B), indole (Figure 3C), 3-indoleacetonitrile, 5-fluoro-2-methylindole, 5-fluoroindole, and 5-methylindole (Supplementary Figure 6) were ranged from 2.5 to 5 mM. These results indicate indoles effectively suppress prodigiosin synthesis and biofilm formation by *S. marcescens* by inhibiting QS activity rather than by exhibiting antimicrobial activity, which suggests indoles may be less prone to the development of drug resistance than conventional antibiotics.

**FIGURE 2:**
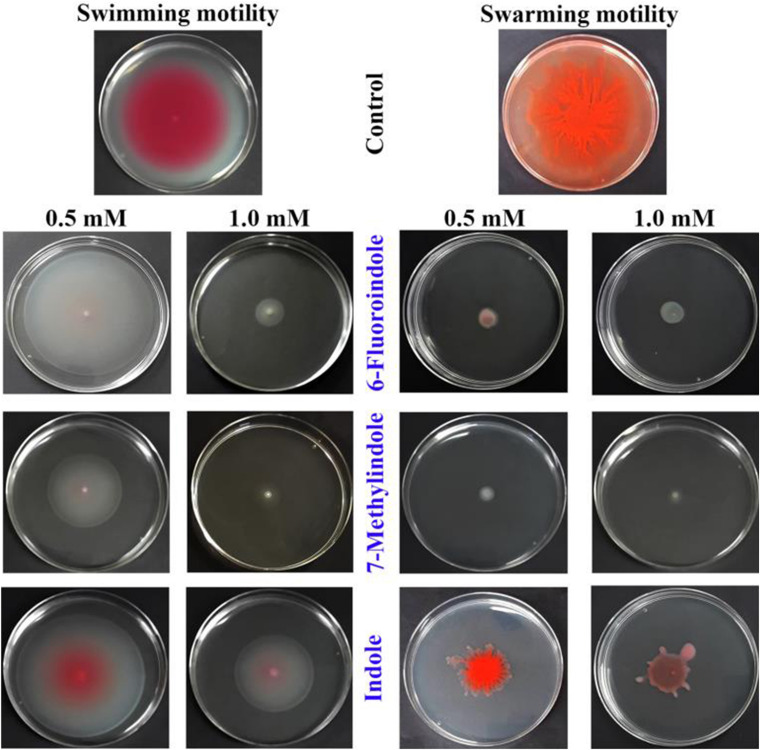
Effects of indole and indole derivatives on the swimming and swarming motilities of *S. marcescens*.

In *S. marcescens*, AHL mediated QS controls swarming and swimming motilities (Horng et al., 2002; Coulthurst et al., 2006). Our results on the swarming and swimming inhibitory activities of indole and the six indole derivatives are presented in Figure 2 and Supplementary Figure 7. Of the indole derivatives tested, 3-indoleacetonitrile, 5-fluoroindole, 6-fluoroindole, 5-methylindole, and 7-methylindole markedly inhibited the swarming motility of *S. marcescens*, whereas indole had a moderate inhibitory effect (Figure 2 and Supplementary Figure 7). As regards the inhibition of swimming motility, 5-fluoroindole, 6-fluoroindole, 5-methylindole, and 7-methylindole were found to be most effective (Figure 2 and Supplementary Figure 7).

### Effects of Indole Derivatives on Protease and Lipase Productions

**FIGURE 3:**
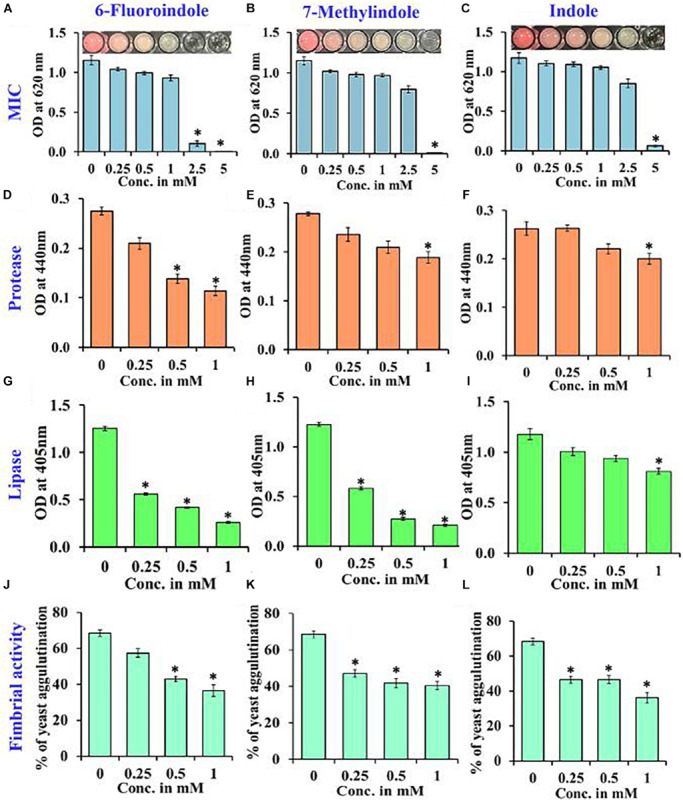
Determination of the MICs of 6-fluoroindole **(A)**, 7-methylindole **(B)**, and indole **(C)** against *S. marcescens.* Effects of 6-fluoroindole, 7-methylindole, and indole on protease **(D–F)**, lipase **(G–I)** levels and on fimbria-mediated yeast agglutination **(J–L)** of *S. marcescens*. Error bars and asterisks (*) represent standard deviations and significant differences (*p* < 0.05), respectively, vs. non-treated controls.

Proteases secreted by bacterial pathogens play important roles in the establishment of infections and in systemic dissemination by degrading host defense proteins (Saint-Criq et al., 2018). Indole and 3-indoleacetonitrile, 5-fluoroindole, 6-fluoroindole, 5-methylindole, and 7-methylindole at 1 mM were found to inhibit extracellular protease production effectively and dose-dependently by 25–60% (Figures 3D–F and Supplementary Figure 8). Among the six indole derivatives tested, 3-indoleacetonitrile, 5-fluoroindole, 6-fluoroindole, 5-fluoro-2-methylindole, and 7-methylindole at a concentration of 1 mM were found to inhibit lipase production by 60–80% (Figures 3G–I and Supplementary Figure 9).

### Effects of Indole Derivatives on Fimbria-Mediated Yeast Agglutination and on Sensitivity to H_2_O_2_

**FIGURE 4:**
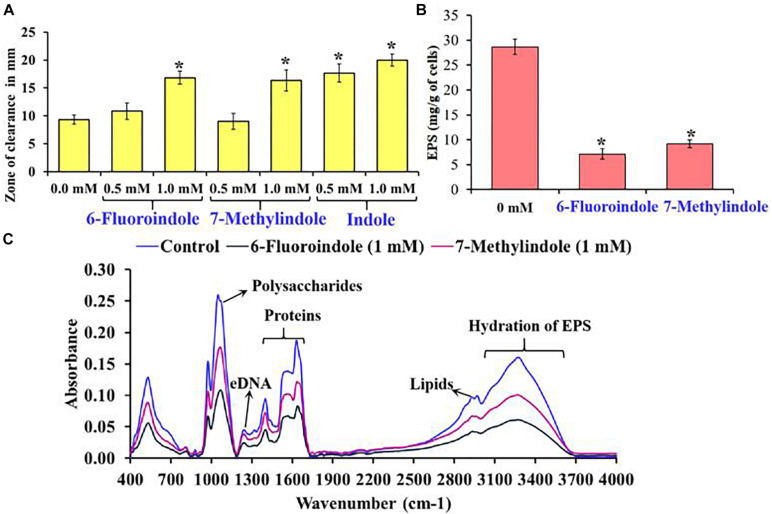
Effects of 6-fluoroindole, 7-methylindole, and indole on the sensitivity of *S. marcescens* to H_2_O_2_ **(A)**. Effects of 6-fluoroindole and 7-methylindole at 1 mM on EPS production by *S. marcescens* **(B)**. Error bars and asterisks (*) represent standard deviation and significant differences (*p* < 0.05), respectively, vs. non-treated controls. FTIR analysis of EPS extracted from control, 6-fluoroindole, and 7-methylindole treated *S. marcescens* **(C)**.

In *S. marcescens*, the transcriptional regulator OxyR is required for the regulation of oxidative stress response and the initial stages of biofilm formation via the modulation of the expression of type I fimbria (Shanks et al., 2007). Furthermore, QS inhibitors such as phenol, 2,4-bis(1,1-dimethylethyl) (Padmavathi et al., 2014), and phytol (Srinivasan et al., 2016) have been shown to inhibit fimbrial expression in *S. marcescens*, and hence, we evaluated the effect of selected indole derivatives on fimbria-mediated yeast agglutination and sensitivity to H_2_O_2_. We found *S. marcescens* cells in the presence of 5-fluoroindole, 6-fluoroindole, 5-fluoro-2-methylindole, 5-methylindole, 7-methylindole, or indole at 1 mM *S. cerevisiae* agglutination by 36–68% (Figures 3J–L and Supplementary Figure 10). Also, we found the sensitivities of 6-fluoroindole, 7-methylindole, or indole treated *S. marcescens* cells to H_2_O_2_ were greater than that of non-treated controls (Figure 4A).

### Effects of 6-Fluoroindole and 7-Methylindole on EPS Production

Bacterial cells constitute 10–20% of wound biofilms, whereas EPS accounts for 80–90% of total biofilm mass (Percival et al., 2014). We observed EPS production was inhibited in 6-fluoroindole or 7-methylindole treated *S. marcescens* (Figure 4B). Extracted EPS was subjected to FTIR, which showed the presence of polysaccharides (1,200–900 cm^–1^), amide I proteins (peaks corresponding to C=O and C-N stretching vibrations at 1,600 and 1,700 cm^–1^), amide II proteins (peaks corresponding to N-H bending and C-N and C-C stretching vibrations at 1,510 and 1,580 cm^–1^), and lipids (signature peaks at 2,850–3,020 cm^–1^) (Naumann, 2001; Badireddy et al., 2008; Figure 4C). Reductions in saccharide, protein, and lipid absorptions showed 6-fluoroindole and 7-methylindole reduced EPS production by *S. marcescens*. In addition, EPS extracted from 6-fluoroindole, and 7-methylindole treated *S. marcescens* showed less hydration than the non-treated control (Figure 4C).

### Differential Expressions of Genes by 6-Fluoroindole in *S. marcescens*

**FIGURE 5:**
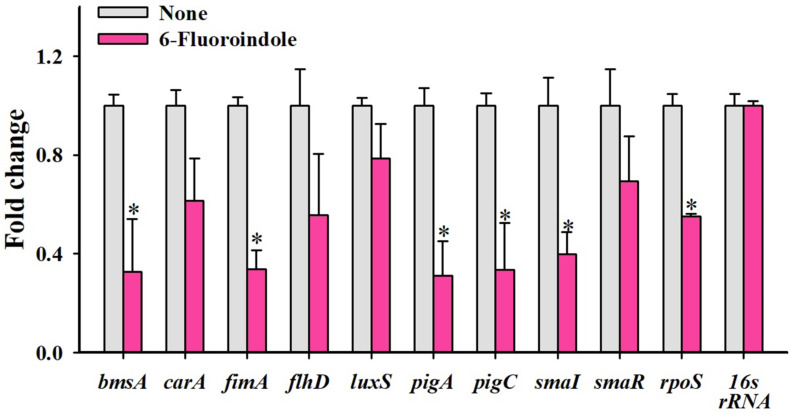
Relative transcriptional profiles of *S. marcescens* cells treated with or without 6-fluoroindole. *S. marcescens* was incubated with or without 6-fluoroindole at 0.5 mM for 6 h with shaking at 250 rpm. Transcriptional profiles were obtained by qRT-PCR. Fold changes represent changes in the transcriptions of treated vs. untreated *S. marcescens*. *16s rRNA* was a housekeeping gene. ^∗^*P* < 0.05 vs. non-treated controls (None).

qRT-PCR was used to investigate the effects of 6-fluoroindole on the expressions of 10 QS-related genes associated with inhibitions of QS and biofilm formation. Notably, six key biofilm-, prodigiosin- and QS-genes, that is, *bmsA* (−3.1 ± 0.2), *fimA* (−3.0 ± 0.1), *pigA* (−3.2 ± 0.1), *pigC* (−3.0 ± 0.2), *Sma*I (−1.4 ± 0.2), and *rpoS* (−1.8 ± 0.1), were significantly repressed by 6-fluoroindole at 0.5 mM (Figure 5). These transcriptomic data partially support the inhibition of prodigiosin production and biofilm formation (Figure 1) and swarming inhibition (Figure 2). Interestingly, *Sma*I was more significantly inhibited than that of *SmaR* and *luxS* by 6-fluoroindole.

### QS Inhibition by Indoles

**FIGURE 6:**
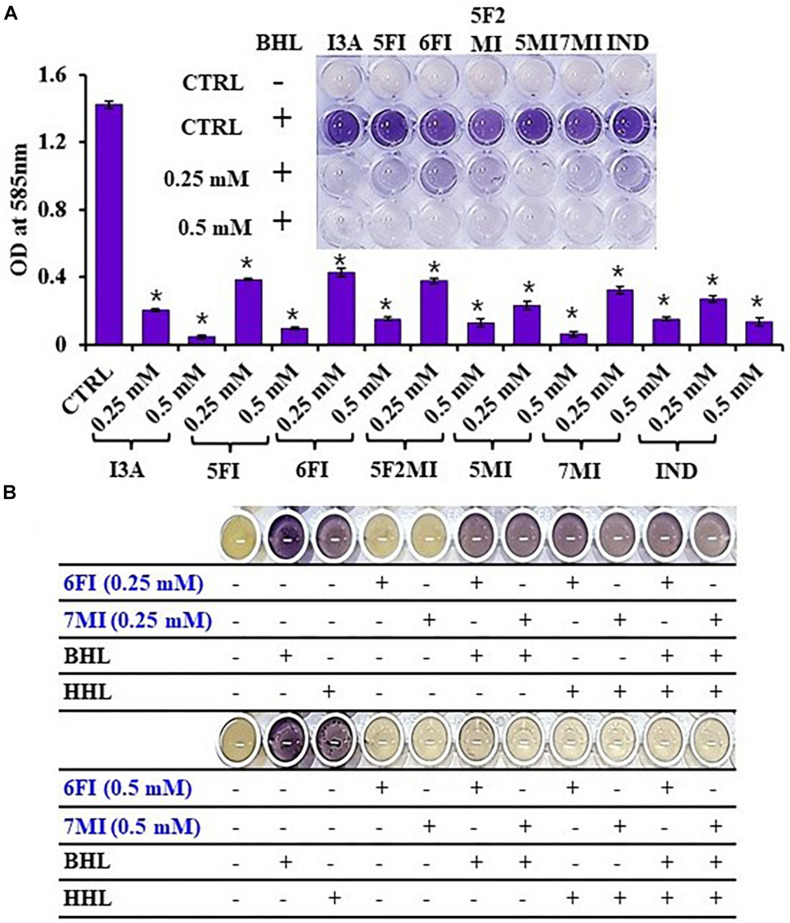
Indole (IND) and selected indole derivatives [3-indoleacetonitrile (I3A), 5-fluoroindole (5FI), 6-fluoroindole (6FI), 5-fluoro-2-methylindole (5F2MI), 5-methylindole (5MI), and 7-methylindole (7MI)] inhibited QS controlled violacein pigment production in *Chromobacterium violaceum* CV026. The inset shows the inhibition of violacein pigment production by indole and selected indole derivatives. Error bars and asterisks (*) represent standard deviations and significant differences (*p* < 0.05), respectively, vs. non-treated controls (CTRL) **(A)**. *N*-butanoyl homoserine lactone (BHL) or *N*-hexanoyl homoserine lactone (HHL) at 500 μM was used for complementing violacein production in the presence of 6-fluoroindole (6FI) and 7-methylindole (7MI) **(B)**.

Reporter strain *C. violaceum* CV026 is widely used as a biosensor strain for the screening of QS inhibitors that lack AHL synthase (CviI), and exogenous AHL supplementation restores QS-mediated violacein pigment production (McClean et al., 1997). We assessed violacein production using CV026 in the presence of exogenous AHL and indole or six indole derivatives (3-indoleacetonitrile, 5-fluoroindole, 6-fluoroindole, 5-fluoro-2-methylindole, 5-methylindole, and 7-methylindole). We found that at 0.25 mM indole and the six indole derivatives markedly inhibited violacein pigment production (Figure 6A), and thus, QS activity. For example, two active 6-fluoroindole and 7-methylindole at 0.25 mM decreased cell growth by only 9 and 24% while QS activity was decreased by 71 and 77% (Supplementary Table 2).

Additionally, the addition of AHLs (BHL or HHL) partially complemented QS inhibition by indoles. For example, BHL or HHL mostly restored QS activity in the presence of 6-fluoroindole (6FI) and 7-methylindole (7MI) at 0.25 mM while two AHLs could not complement the presence of the higher amount of two indoles at 0.5 mM (Figure 6B). This result also supports the previous finding that violacein inhibition by indole could be counteracted by the exogenous C10-AHL (Hidalgo-Romano et al., 2014).

## Discussion

Several phenolics are known to inhibit prodigiosin synthesis by *S. marcescens* (Kalia, 2013). Indole and its derivatives are synthesized by various microbes as signaling molecules that control various aspects of bacterial and eukaryotic physiology (Lee and Lee, 2010; Lee et al., 2015b). These interspecies and interkingdom signaling molecules inhibit QS, biofilm formation, and the expressions of virulence factors in non-indole producing bacteria (Kalia, 2013; Lee et al., 2015b). The current study shows that indoles have diverse antivirulence roles in *S. marcescens*. Like indoles, furocoumarins (Girennavar et al., 2008), coumarins (Gutiérrez-Barranquero et al., 2015; D’Almeida et al., 2017), 6,7-dihydroxycoumarin, and 7-hydroxycoumarin (Ta and Arnason, 2016) inhibit AHL-mediated QS and biofilm formation.

The *pig*-gene cluster encodes for a group of enzymes responsible for the biosynthesis of prodigiosin in *S. marcescens*, and the majority of these genes are involved in the conversion of 2-octenal to monopyrrole MAP (2-methyl-3-amylpyrrole) and the bipyrrole moiety MBC (4-methoxy-2-2′-bipyrrole-5-carbaldehyde). *pigC* encodes for an enzyme that condenses MBC and MAP to produce prodigiosin (Pan et al., 2020). A significant reduction in prodigiosin levels was observed after exposing with the QS inhibitor hordenine, which down-regulated the expressions of *pig*-genes (Zhou et al., 2019). The expression of the *pig*-gene cluster is also affected by various transcriptional factors (Pan et al., 2020), and is a potential target of indole derivatives.

Indole and its derivatives are viewed as potential antivirulence compounds against antibiotic-resistant pathogens because of their ability to inhibit quorum sensing and virulence factor production (Lee et al., 2015b). Interestingly these indoles affect bacterial physiology in different ways. For example, indoles activate efflux pump systems in *Escherichia* sp. (Kawamura-Sato et al., 1999; Lee et al., 2010), *Vibrio* sp. (Howard et al., 2019), *Pseudomonas* sp. (Lee et al., 2009, 2012; Molina-Santiago et al., 2014), *Agrobacterium* sp. (Lee et al., 2015a), *Cylindrotheca* sp. (Yang et al., 2014), and *Salmonella* sp. (Nikaido et al., 2008, 2012; Blair et al., 2013) and inhibit QS systems in *Pseudomonas* sp. (Lee et al., 2009, 2011, 2012; Tashiro et al., 2010; Chu et al., 2012; Frei et al., 2012; Biswas et al., 2015), *Acinetobacter* sp. (Kim and Park, 2013), and *Chromobacterium* sp. and *Serratia* sp. (Hidalgo-Romano et al., 2014). Although the exact mechanisms responsible for the effects of indoles have not been determined (Kim and Park, 2015; Lee et al., 2015b; Zarkan et al., 2020), the abundance of indole derivatives presents an opportunity to identify indoles active against super bacteria.

Bacterial swarming involves the well-coordinated migration of cells driven by flagella and plays an important role in nutrient sensing, surface colonization, biofilm formation, virulence, and host-pathogen interactions (Kearns, 2010). In addition, swarming motility is associated with the resistance to antimicrobial agents displayed by several clinically important pathogens (Kearns, 2010). Thus, compounds that diminish swarming motility are likely to affect biofilm formation and virulence factor production (Corral et al., 2020; Rütschlin and Böttcher, 2020). Similarly, swimming motility is also involved in the initial phase of the infection process (Kumar et al., 2018; Corral et al., 2020). In this study, several indoles significantly inhibit swarming and swimming motilities (Figure 2 and Supplementary Figure 7).

Therapeutic agents that reduce the virulence of pathogens are topics of active research in the pharmaceutical industry and in academia. Protease defective strains of *P. aeruginosa* (Breidenstein et al., 2012) and *Vibrio cholera* (Rogers et al., 2016) reduce motility, biofilm formation, and virulence, and bioactive compounds like N-mercaptoacetyl-Phe-Tyr-amide (Cathcart et al., 2011), curcumin (Rudrappa and Bais, 2008; Sethupathy et al., 2016a), and hydroxamic acid (Kany et al., 2018) have been reported to reduce the virulence and biofilm formation by *P. aeruginosa* by targeting protease. Lipases are important secreted virulence factors that support bacterial and fungal pathogens during the early infection stage by damaging the phospholipid layers of host cells and disrupting innate defenses (Chen and Alonzo, 2019). Bioactive compounds such as alpha-bisabolol (Sethupathy et al., 2016b), vanillic acid (Sethupathy et al., 2017), and phytol (Srinivasan et al., 2016) have been shown to affect protease and lipase production in *S. marcescens* by interfering with QS. The protease and lipase inhibitory activities of indole derivatives against *S. marcescens* (Figure 3 and Supplementary Figures 8, 9) warrant further investigation as potential therapeutic agents, and the results of our H_2_O_2_ sensitivity assay also suggest the disruption of oxidative stress response in *S. marcescens*, which is one of the prerequisites of biofilm formation under challenging conditions *in vivo*. Our findings indicate that both 6-fluoroindole and 7-methylindole reduce yeast agglutination and increase sensitivity to H_2_O_2_ probably through the differential regulation of OxyR in *S. marcescens*.

Previously, the addition of exogenous C10-AHL could restore the production of QS activity (Hidalgo-Romano et al., 2014) and the current studies also showed that the additions of other AHLs (BHL or HHL) mostly restored QS activity in the presence of indole derivatives (Figure 6B). Similarly, 6-gingerol (Kim H.-S. et al., 2015) and quercetin (Gopu et al., 2015) were reported to inhibit violacein production in the presence of AHL by blocking AHL-transcriptional receptor protein complex, which is essential for the violacein biosynthesis. In the majority of Gram-negative pathogens, AHL-transcriptional receptor protein complex formation is essentially required for the activation of QS controlled phenotypes and virulence gene expression (Miller and Bassler, 2001; Mukherjee and Bassler, 2019) and unlike antimicrobial agents, inhibiting AHL-transcriptional receptor protein complex formation by indoles is expected to reduce pressure favoring the development of drug resistance.

While indole and most of the indole derivatives did not have significant antimicrobial activity in *S. marcescens* (Figure 1 and Supplementary Figure 3), a few indoles such as 5-fluoroindole, 5-iodoindole and 5-methylindole showed significant antimicrobial activity (Supplementary Figure 4). Also, in the *C. violaceum* strain, most indoles at 0.25 and 0.5 mM markedly affect planktonic cell growth (Supplementary Table 2). Furthermore, it was reported that 5-iodoindole showed strong bactericidal activity against *Escherichia coli* strains and *Staphylococcus aureus* (Lee et al., 2016) and could rapidly kill *Acinetobacter baumannii* (Raorane et al., 2020). Therefore, it is important to carefully assess the toxicity of indoles on bacteria and animals.

The present study demonstrates the abilities of indole derivatives to inhibit QS in *S. marcescens*, and thus, to inhibit prodigiosin pigment production, biofilm formation, swimming motility, swarming motility, and fimbrial activity. Of the indole derivatives tested, 6-fluoroindole and 7-methylindole potently inhibited lipase, protease, and EPS production in *S. marcescens*. AHL supplementation assay using *C. violaceum* CV026 confirmed the disruption of QS by indole derivatives. Based on the results obtained, we suggest that indole and indole derivatives interfere with QS in *S. marcescens* and *C. violaceum* possibly by preventing AHL molecules binding to their cognate receptors. Based on our observation that indole derivatives differentially inhibit the virulence of and biofilm formation by *S. marcescens*, we suggest further studies be undertaken to determine the molecular mechanism involved.

## Data Availability Statement

All datasets generated for this study are included in the article/Supplementary Materials, further inquiries can be directed to the corresponding authors.

## Author Contributions

SS, J-HL, and JL: conceptualization. SS, ES, Y-GK, and JL: methodology. SS, ES, J-HL, Y-GK, and JL: writing of the original manuscript. J-HL and JL: project administration and funding acquisition. All authors contributed to the article and approved the submitted version.

## Conflict of Interest

The authors declare that the research was conducted in the absence of any commercial or financial relationships that could be construed as a potential conflict of interest.

## Supplementary Material

Supplementary Figure 1 Screening of indole derivatives for the QS controlled inhibition of prodigiosin production in *S. marcescens* [Indole-3-carboxaldehyde (1), indole-3-butyric acid (2), indole-3-carbinol (3), indole-3-acetic acid (4), indole-3-carboxylic acid (5), 2-oxindole (6), 7-methoxyindole (7), 5-fluoroxindole (8), 7-fluoroindole-2,3-dione (9), 3-indoleacetonitrile (10), 5-fluoroindole (11), 6-fluoroindole (12), 7-fluoroindole (13), 5-fluoroindole-2,3-dione (14), 3,3’-methylenebis-indole (15), methyl-indole-7-carboxylate (16), 2,5-dimethylindole (17), 7-methylindole (18), 7-methyl-1H-indole-2,3-dione (19), 4-formyl indole (20), 5-formyl indole (21), 6-formyl indole (22), 7-formyl indole (23), 7-nitroindole (24), 7-azaindole (25), 6-(trifluoromethyl)indole (26), 1H-indole-2-carboxaldehyde (27), 5-iodoindole (28), 2-methylindole-3-acetic acid (29), 5-amino-2-methylindole (30), 5-indoindolin-2-one (31), 5-fluoro-2-methylindole (32), 5-chloro-2-methylindole (33), 7-fluoro-5-iodoindole (34), indole-3-acetamide (35), indole-3-propionic acid (36), indole-7-carboxylic acid (37), 1-methylindole-3-carboxaldehyde (38), 7-methylindole-3-carboxaldehyde (39), 2-methylindole-3-carboxaldehyde (40), 5-benzyloxyindole (41), 1,2-dimethylindole (42), 5-methylindole (43), 4-benzyloxyindole (44), 6-benzyloxyindole (45), 7-benzyloxyindole (46), 7-fluoro-5-iodoindole-3-carboxaldehyde (47), 1-BOC-5-iodoindole (48), 3-(2-hydroxyethyl)indole (49), 4-fluoroindole (50), 7-fluoroindoline-2, 3-dione (51), and indole (52)]. Error bars and asterisks represent standard deviation and the statistically significant difference (*p* < 0.05), respectively.

Supplementary Figure 2 Concentration-dependent inhibition of prodigiosin production by selected indole derivatives in *S. marcescens*. Error bars and asterisks represent standard deviation and the statistically significant difference (*p* < 0.05), respectively.

Supplementary Figure 3 Screening of indole and indole derivatives for *S. marcescens* antibiofilm activity. Error bars and asterisks represent standard deviation and the statistically significant difference (*p* < 0.05), respectively. Tested indole derivatives names are indicated in Supplementary Figure 1.  Biofilm formation  Planktonic cell growth.

Supplementary Figure 4 Concentration-dependent antibiofilm activities of selected indole derivatives against *S. marcescens*. Error bars and asterisks represent standard deviation and the statistically significant difference (*p* < 0.05), respectively.  Biofilm formation  Planktonic cell growth

Supplementary Figure 5 Effects of indole derivatives on the planktonic cell growth of *S. marcescens*.

Supplementary Figure 6 Determination of the MICs of 3-indoleacetonitrile, 5-fluoroindole, 7-methylindole, and 5-fluoro-2-methylindole against *S. marcescens.* Error bars and asterisks represent standard deviation and the statistically significant difference (*p* < 0.05), respectively.

Supplementary Figure 7 Inhibitory effects of indole derivatives on the swimming and swarming motilities of *S. marcescens.*

Supplementary Figure 8 Effects of indole derivatives on protease production by *S. marcescens.* Error bars and asterisks represent standard deviation and the statistically significant difference (*p* < 0.05), respectively.

Supplementary Figure 9 Effects of indole derivatives on lipase production in *S. marcescens.* Error bars and asterisks represent standard deviation and the statistically significant difference (*p* < 0.05), respectively.

Supplementary Figure 10 Effects of indole derivatives on the fimbria-mediated yeast agglutination by *S. marcescens.* Error bars and asterisks represent standard deviation and the statistically significant difference (*p* < 0.05), respectively.

Supplementary Table 1 Primer sequences for qRT-PCR.

Supplementary Table 2 Comparison of QS inhibition and growth inhibition by indoles in *C. violaceum* CV026. This table supports the Figure 6 of QS inhibition assay.
