# Supplementary material for: Antibiofilm and Antivirulence Properties of Indoles Against Serratia marcescens
Source: Front Microbiol. 2020 Oct 30;11:584812. doi: 10.3389/fmicb.2020.584812 (PMC7662412; doi:10.3389/fmicb.2020.584812)
Supplement: Supplementary Figure 1 — Screening of indole derivatives for the QS controlled inhibition of prodigiosin production in S. marcescens [Indole-3-carboxaldehyde (1), indole-3-butyric acid (2), indole-3-carbinol (3), indole-3-acetic acid (4), indole-3-carboxylic acid (5), 2-oxindole (6), 7-methoxyindole (7), 5-fluoroxindole (8), 7-fluoroindole-2,3-dione (9), 3-indoleacetonitrile (10), 5-fluoroindole (11), 6-fluoroindole (12), 7-fluoroindole (13), 5-fluoroindole-2,3-dione (14), 3,3’-methylenebis-indole (15), methyl-indole-7-carboxylate (16), 2,5-dimethylindole (17), 7-methylindole (18), 7-methyl-1H-indole-2,3-dione (19), 4-formyl indole (20), 5-formyl indole (21), 6-formyl indole (22), 7-formyl indole (23), 7-nitroindole (24), 7-azaindole (25), 6-(trifluoromethyl)indole (26), 1H-indole-2-carboxaldehyde (27), 5-iodoindole (28), 2-methylindole-3-acetic acid (29), 5-amino-2-methylindole (30), 5-indoindolin-2-one (31), 5-fluoro-2-methylindole (32), 5-chloro-2-methylindole (33), 7-fluoro-5-iodoindole (34), indole-3-acetamide (35), indole-3-propionic acid (36), indole-7-carboxylic acid (37), 1-methylindole-3-carboxaldehyde (38), 7-methylindole-3-carboxaldehyde (39), 2-methylindole-3-carboxaldehyde (40), 5-benzyloxyindole (41), 1,2-dimethylindole (42), 5-methylindole (43), 4-benzyloxyindole (44), 6-benzyloxyindole (45), 7-benzyloxyindole (46), 7-fluoro-5-iodoindole-3-carboxaldehyde (47), 1-BOC-5-iodoindole (48), 3-(2-hydroxyethyl)indole (49), 4-fluoroindole (50), 7-fluoroindoline-2, 3-dione (51), and indole (52)]. Error bars and asterisks represent standard deviation and the statistically significant difference (p < 0.05), respectively. [file Data_Sheet_1.docx]

**Supplementary information**

**Antibiofilm and antivirulence properties of indoles against *Serratia marcescens***

Sivasamy Sethupathy^§^, Ezhaveni Sathiyamoorthi^§^, Yong-Guy Kim, Jin-Hyung Lee*, and Jintae Lee*

School of Chemical Engineering, Yeungnam University, 280 Daehak-Ro, Gyeongsan 38541, Republic of Korea

^§^These authors contributed equally to this work.

*Corresponding Authors

E-mail: jinhlee@ynu.ac.kr. Tel.: +82-53-810-3812. Fax: +82-53-810-4631.

E-mail: jtlee@ynu.ac.kr. Tel.: +82-53-810-2533. Fax: +82-53-810-4631.

**Supplementary Table 1. Primer sequences for qRT-PCR**

| **Gene** | **Function** | **Primer** |
| --- | --- | --- |
| *bmsA* | Biofilm formation | Forward 5'-CGGAAGTGACGCTGGAACACG-3' |
|  |  | Reverse 5'-TGCTGCTGTTGATGGTGTAATCGG-3' |
| *carA* | Glutamine-hydrolyzing carbamoyl-phosphate synthase small subunit; prodigiosin biosynthesis | Forward 5'-GGAGAATTCTGCTTCTTGATTG-3' |
|  |  | Reverse 5'-CTGACAAACCCGATCTTCAC-3' |
| *fimA* | Type 1 fimbrial protein; fimbriae production | Forward 5'-TTAGCCTGGAGAAATGTGAAGC-3' |
|  |  | Reverse 5'-GGCAGAGTAGAGCCGTTGTTAT-3' |
| *flhD* | Flagellar transcriptional regulator FlhD; motility | Forward 5'-CCTCCGCGATGTTCCGTCTTG-3' |
|  |  | Reverse 5'-GGTCAGGCGTTCGATGGTCTG-3' |
| *luxS* | S-ribosylhomocysteine lyase; AI-2-dependent quorum sensing | Forward 5'-TGGCGTGGAAGTTGTTGATA-3' |
|  |  | Reverse 5'-CATTGAGCTCCGGGATTTTA-3' |
| *pigA* | Prodigiosin synthetic enzyme; prodigiosin production | Forward 5'-ATGGCTTTATGGGCGTGTC-3' |
|  |  | Reverse 5'-TGAAGGTCAGTTCGCTCCAC-3' |
| *pigC* | Prodigiosin synthetic enzyme; prodigiosin production | Forward 5'-TTCGTCACAAACCGCACTATT-3' |
|  |  | Reverse 5'-CGTCTTTCACCGCCCATT-3' |
| *smaI* | DNA-binding transcriptional regulator; LuxIR-type quorum sensing system | Forward 5'-TGCCTGTGGATGAGCATAAC-3' |
|  |  | Reverse 5'-TCCCTGACGCAGAATGATAG-3' |
| *smaR* | DNA-binding transcriptional regulator; LuxIR-type quorum sensing system | Forward 5'-GACAAATCCTGCGATGATGA-3' |
|  |  | Reverse 5'-CCATGCTTGCCCAGTAAAGT-3' |
| *rpoS* | RNA polymerase sigma factor; a pleiotropic regulator of motility, biofilm formation, exoenzymes, siderophore and prodigiosin production | Forward 5'-AGGATTCCGCTTTTCAACCT-3’ |
|  |  | Reverse 5'-CTATCGAGCTGTTCGGCAAT-3’ |
| *16S rRNA* | Housekeeping | Forward 5'-GGCCTTCGGGTTGTAAAGTC-3’ |
|  |  | Reverse 5'-GCTTTACGCCCAGTCATTC-3’ |

**Supplementary Table 2. Comparison of QS inhibition and growth inhibition by indoles in *C. violaceum* CV026.** This table supports the Figure 6 of QS inhibition assay.

| Compounds | QS inhibition at 0.25 mM (%) | QS inhibition at 0.5 mM (%) | Growth inhibition at 0.25 mM (%) | Growth inhibition at 0.5 mM (%) |
| --- | --- | --- | --- | --- |
| 3-Indoleacetonitrile | 85.6 | 97.0 | 30.8 | 73.4 |
| 5-Fluoroindole | 72.7 | 93.5 | 14.4 | 71.0 |
| 6-Fluoroindole | 70.7 | 89.6 | 9.3 | 53.2 |
| 5-Fluoro-2-methylindole | 73.7 | 91.0 | 25.0 | 71.3 |
| 5-Methylindole | 83.6 | 95.6 | 27.12 | 73.7 |
| 7-Methylindole | 77.4 | 89.3 | 24.1 | 35.9 |
| Indole | 81.1 | 90.6 | 11.7 | 20.7 |

**Supplementary Figure 1.** Screening of indole derivatives for the QS controlled inhibition of prodigiosin production in *S. marcescens*. (Indole-3-carboxaldehyde **(1)**, indole-3-butyric acid **(2)**, indole-3-carbinol **(3)**, indole-3-acetic acid **(4)**, indole-3-carboxylic acid **(5)**, 2-oxindole **(6)**, 7-methoxyindole **(7)**, 5-fluoroxindole **(8)**, 7-fluoroindole-2,3-dione **(9)**, 3-indoleacetonitrile **(10)**, 5-fluoroindole **(11)**, 6-fluoroindole **(12)**, 7-fluoroindole **(13)**, 5-fluoroindole-2,3-dione **(14)**, 3,3'-methylenebis-indole **(15)**, methyl-indole-7-carboxylate **(16)**, 2,5-dimethylindole **(17)**, 7-methylindole **(18)**, 7-methyl-1H-indole-2,3-dione **(19)**, 4-formyl indole **(20)**, 5-formyl indole **(21)**, 6-formyl indole **(22)**, 7-formyl indole **(23)**, 7-nitroindole **(24)**, 7-azaindole **(25)**, 6-(trifluoromethyl)indole **(26)**, 1H-indole-2-carboxaldehyde **(27)**, 5-iodoindole **(28)**, 2-methylindole-3-acetic acid **(29)**, 5-amino-2-methylindole **(30)**, 5-indoindolin-2-one **(31)**, 5-fluoro-2-methylindole **(32)**, 5-chloro-2-methylindole **(33)**, 7-fluoro-5-iodoindole **(34)**, indole-3-acetamide **(35)**, indole-3-propionic acid **(36)**, indole-7-carboxylic acid **(37)**, 1-methylindole-3-carboxaldehyde **(38)**, 7-methylindole-3-carboxaldehyde **(39)**, 2-methylindole-3-carboxaldehyde **(40)**, 5-benzyloxyindole **(41)**, 1,2-dimethylindole **(42)**, 5-methylindole **(43)**, 4-benzyloxyindole **(44)**, 6-benzyloxyindole **(45)**, 7-benzyloxyindole **(46)**, 7-fluoro-5-iodoindole-3-carboxaldehyde **(47)**, 1-BOC-5-iodoindole **(48)**, 3-(2-hydroxyethyl)indole **(49)**, 4-fluoroindole **(50),** 7-fluoroindoline-2, 3-dione **(51),** and indole **(52)).** Error bars and asterisks represent standard deviation and the statistically significant difference (*p* < 0.05), respectively.

**Supplementary Figure 2.** Concentration-dependent inhibition of prodigiosin production by selected indole derivatives in *S. marcescens*. Error bars and asterisks represent standard deviation and the statistically significant difference (*p* < 0.05), respectively.

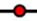
**Supplementary Figure 3.** Screening of indole and indole derivatives for *S. marcescens* antibiofilm activity. Error bars and asterisks represent standard deviation and the statistically significant difference (*p* < 0.05), respectively. Tested indole derivatives names are indicated in Supplementary Figure 1.
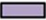
 Biofilm formation Planktonic cell growth

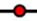
**Supplementary Figure 4.** Concentration-dependent antibiofilm activities of selected indole derivatives against *S. marcescens*. Error bars and asterisks represent standard deviation and the statistically significant difference (*p* < 0.05), respectively.
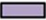
 Biofilm formation Planktonic cell growth

**Supplementary Figure 5.** Effects of indole derivatives on the planktonic cell growth of *S. marcescens*.

**Supplementary Figure 6.** Determination of the MICs of 3-indoleacetonitrile, 5-fluoroindole, 7-methylindole, and 5-fluoro-2-methylindole against *S. marcescens.* Error bars and asterisks represent standard deviation and the statistically significant difference (*p* < 0.05), respectively.

*
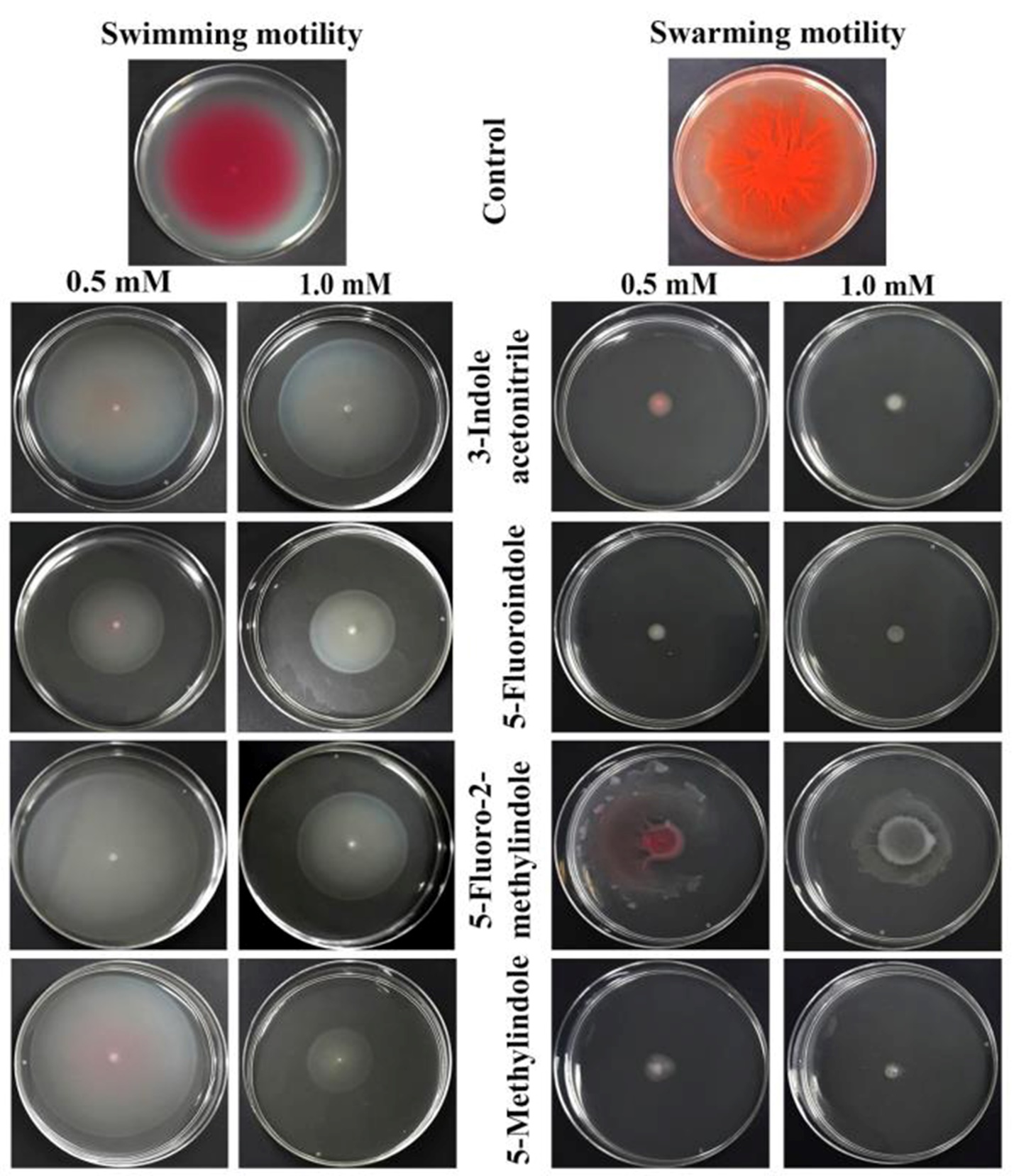
*

**Supplementary Figure 7.** Inhibitory effects of indole derivatives on the swimming and swarming motilities of *S. marcescens.*

**Supplementary Figure 8.** Effects of indole derivatives on protease production by *S. marcescens.* Error bars and asterisks represent standard deviation and the statistically significant difference (*p* < 0.05), respectively.

**Supplementary Figure 9.** Effects of indole derivatives on lipase production in *S. marcescens.* Error bars and asterisks represent standard deviation and the statistically significant difference (*p* < 0.05), respectively.

**Supplementary Figure 10.** Effects of indole derivatives on the fimbria-mediated yeast agglutination by *S. marcescens.* Error bars and asterisks represent standard deviation and the statistically significant difference (*p* < 0.05), respectively.
